# Supplementary material for: Evolution of Resistance to Irinotecan in Cancer Cells Involves Generation of Topoisomerase-Guided Mutations in Non-Coding Genome That Reduce the Chances of DNA Breaks
Source: Int J Mol Sci. 2023 May 13;24(10):8717. doi: 10.3390/ijms24108717 (PMC10218574; doi:10.3390/ijms24108717)
Supplement: Supplementary file 1 [file ijms-24-08717-s001.zip › Supplementary section.pdf]

Supplementary section

Evolution of Resistance to Irinotecan in Cancer Cells Involves Generation of Topoisomerase-Guided Mutations in Non-Coding Genome That Reduce the Chances of DNA Breaks

Santosh Kumar [1], Valid Gahramanov [1], Shivani Patel [1], Julia Yaglom [1], Lukasz Kaczmarczyk [1], Ivan A. Alexandrov [2], Gabi Gerlitz [1], Mali Salmon-Divon [3] and Michael Y. Sherman [1,*]

1 Department of Molecular Biology, Faculty of Natural Sciences, Ariel University, Ariel 40700, Israel; shashisantosh2007@gmail.com (S.K.)
2 Department of Anatomy and Anthropology & Department of Human Molecular Genetics and Biochemistry, Sackler Faculty of Medicine, Tel Aviv University, Tel Aviv 6997801, Israel
3 Adelson School of Medicine, Ariel University, Ariel 40700, Israel
* Correspondence: sherma1@ariel.ac.il

Mutational landscape in SN-38 resistant clones.

As noted in the main text, mutations in coding and regulatory regions did not affect genes involved in cell sensitivity to SN-38. The only exception was a mutation in the MMS22L gene that normally works in the base excision repair, but also plays a role in the DSB repair (Piwko *et al*, 2011; Saredi *et al*, 2016). However, this mutation was a 1 nucleotide insertion, which most likely inactivated the MMS22L gene. In such a scenario, we expect to see sensitization rather than protection from DSBs. Accordingly, it is unlikely that this mutation associates with adaptation to irinotecan (SN-38).

To understand the relevance of the mutations to the adaptation process, we manually identified the precise positions of breaks in 1,000 out of 2,829 NHEJ-repaired common mutation sites and analyzed if there are any shared features in sequences of the sites. About 80% of these breaks took place either within or at the edge of 1-4 bp repeat clusters, and the length of these repeats usually was within the range of 5-30bp (see examples of such repeats in Table S17, Table S18). Though we could not define the breaking points in HR repaired sites precisely, we observed that 75% of these sites are in similar repeats as NHEJ breaking points, and therefore assumed that breaks in these sites occurred in or at the edges of such repeats, as with NHEJ. Overall UCSC analysis showed that the mutations were mostly located in the repetitive elements such as microsatellites (Table S22), simple repeats (Table S23) and satellites (Table S24 for mutations in chromosomal arms and supplement (Table S25) for mutations in pericentromeric genomic locations). Parallel RepeatMasker analysis supports these findings (Figure S4A, Table S26 and S27 for detailed data on repetitive elements and mutation

correlation) where specific satellites such as alpha satellites and human satellite-II is shown to have majority of common mutations (Fig S4B). In other words, repetitive elements accommodate majority of all mutations, including common mutations between the clones, during the adaptation process. Upon analysis of distribution of mutations along the chromosomes, we found that there was a disproportionally around 40% of the total mutations densely populated regions adjusted to centromere (up to 5Mb depending on the chromosome) at both sides (Figure S5), which we referred to as "pericentromeric" areas. This region is identified generally by signatures of H3K9me3 and H3K27me3 followed by richness of repetitive elements such as satellites and simple repeats (Figure S5A, B and Fig S6). As a representative example, Figure S5A shows correlation between pericentromeric locations of common mutations on chromosome-1, pericentromeric chromatin modification signatures, and locations of satellites. For comparison, Fig. S5B shows these distributions and parameters in a chromosomal arm of same chromosome-1. Whereas, Fig. S6 visualizes the overall distribution of common mutations in genome and show its specific enriched presence in centromere and pericentromeric regions.

A very common sequence at the breaking points in the chromosome arms was a polyC stretch (25% of all mutation sites in the arms). The length of these polyC sequences associated with breaking points usually were within the range of 20-40bp. To our surprise, in 100% cases of LOH in these regions, original long polyC was substituted with polyC stretch that was interrupted by multiple SNPs (Figure S9). The fact that these breaks were generated in response to Top1 inhibition by SN-38 suggests that Top1 either binds to such sites specifically, or stalls there in the process of its function, or is activated at these sites.

Supplement figures

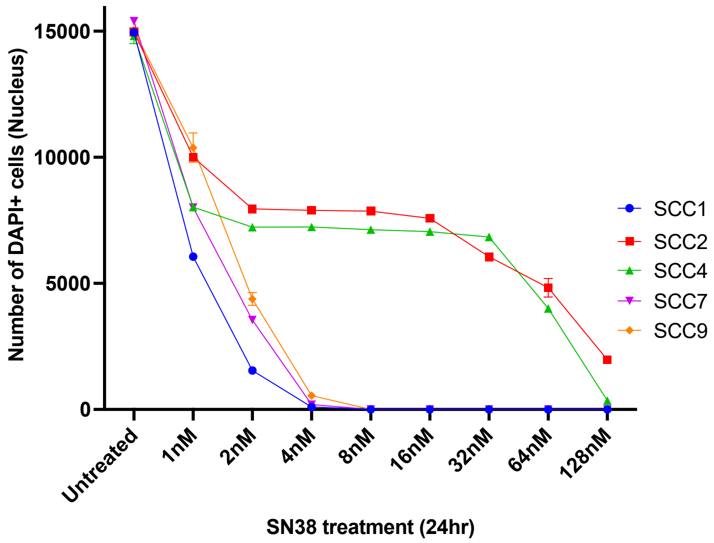

Figure S1. Sensitivity of the single cell clones to SN-38: Isolated clones were grown individually on 24 well plates followed by treatment with several concentrations of SN-38 and were incubated for different time durations for assessment of cell death. After incubation, dead cells were washed and attached cells were fixed, followed by DAPI staining. Using Hermes imaging system, images (n=244) were taken for each well. Experiment was conducted in biological triplicates (n=3) to quantify the cell cytotoxicity.

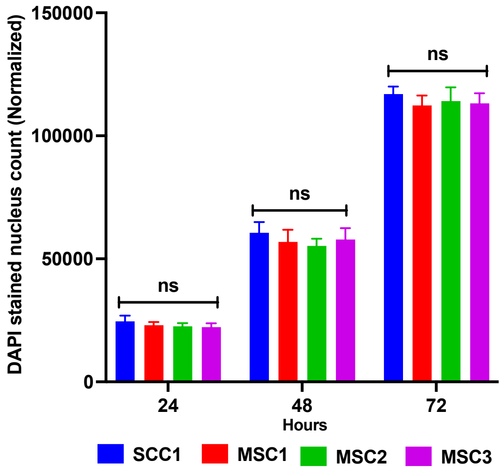

Figure S2. Growth rate of parental clone and resistant mutants: Equivalent number of parental clone population (SCC1) and resistant mutants were plated and kept for normal growing. At time 24-hour interval, cells were fixed and stained by DAPI. Imaging was performed by Hermes imaging system where n=268 images were taken per well; experiment was performed with six biological replicates (n=6). Data shows non-significant difference in increase of cell population among parental clone or mutants indicating the growth rate of

parental and mutant are similar in culture. Statistics were calculated using GraphPad Prism version 9.0.0, California USA. The significance of differences was determined by Ordinary one-way ANOVA, error bar represents standard deviation (computed P=0.2009 is “ns”) as denoted indicating that there was no significant difference at same time points in SCC1 or resistant mutant growth.

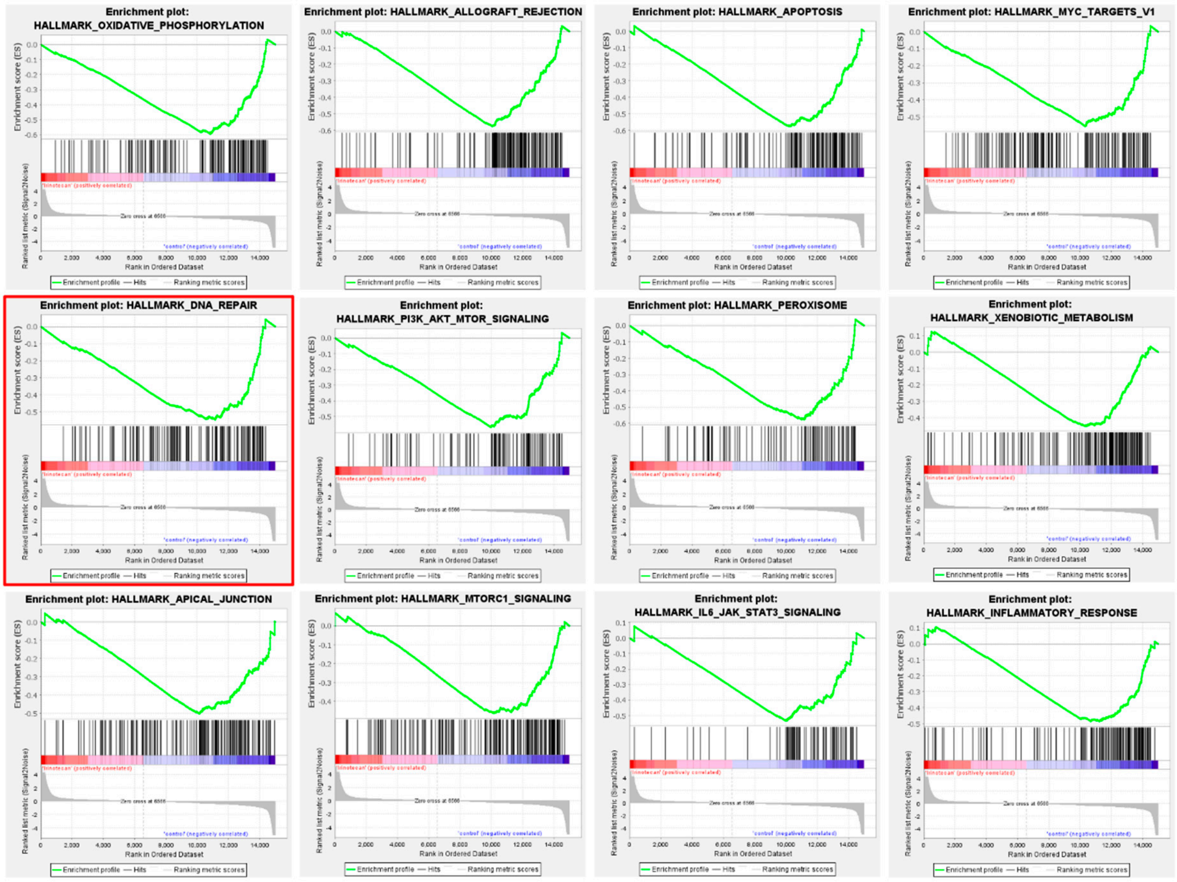

Figure S3. Top-10 enriched pathways uncovered from shRNA screen. A representative plot obtained from GSEA analysis exhibiting important sensitizer pathways (neg-regulated) in response to SN-38, **Top horizontal panel:** Showing Oxidative phosphorylation, Allograft rejection, Apoptosis, Myc. **Middle horizontal panel:** DNA repair (important for survival considering drug favors DNA breaks, marked in red box), MTOR, Peroxisome, and Xenobiotic metabolism (includes MDR’s and drug metabolism related genes), **Lower horizontal panel:** Apical junction, MTORC1, IL6_JAK_STAT3, Inflammatory response. These pathways are top-12 pathways with highly significant False discovery rate (FDR).

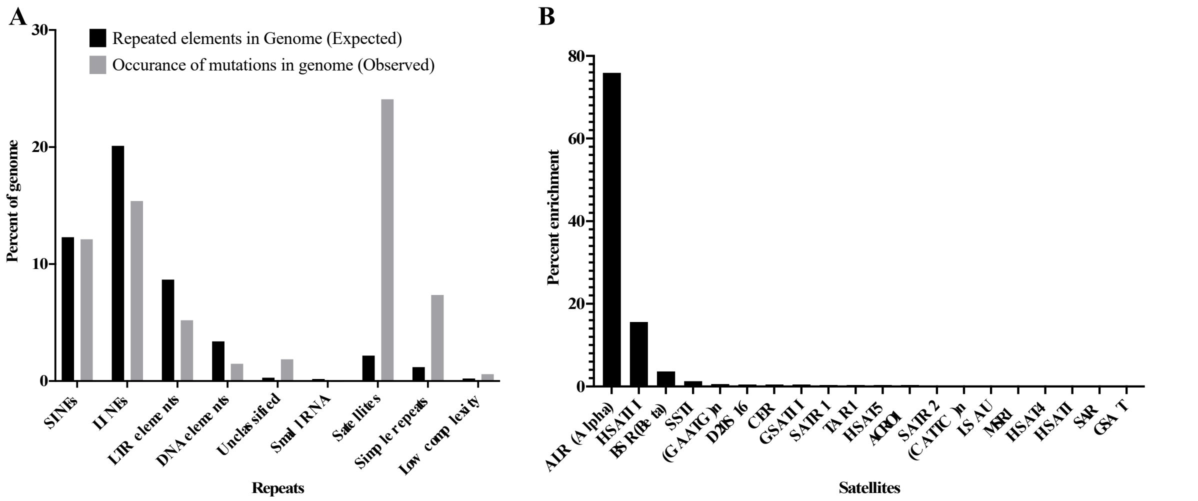

Figure S4. (A) Distribution of repeated elements in human genome (Expected) and fraction of mutations in MSC clones found in these repeats. common mutation sites were taken randomly (100bp upstream and downstream of SNP's) and run through the RepeatMasker (v4.1.0) using nhmmscan version 3.3.1 (Jul 2020). The presence of mutations in SINE corresponds to the presence of SINE in the genome, indicating lack of the enrichment. Strong enrichment was found in satellites and simple repeats, **(B)** In-depth analysis for classification of satellites shows vast majority of common mutations are present in ALR and human satellite II followed by BSR type in all three clones.

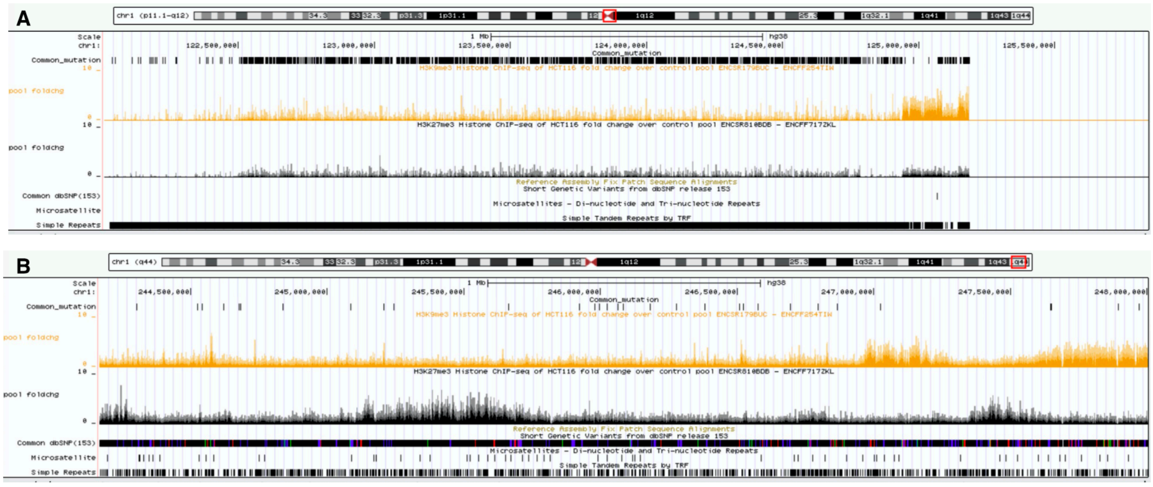

Figure S5. Representative example for pericentromeric and chromosomal arm: **(A)** plot shows correlation between common mutations in "pericentromeric region" marked by H3K9me3 and H3K27me3, where mutations are densely accommodated and covered by simple repeats.

Mutations in these genomic locations are not common dbSNP's and is accommodated by fewer microsatellites, **(B)** shows arm portion of chromosome with comparatively much fewer common mutations and scattered simple repeats in sliding window UCSC browser.

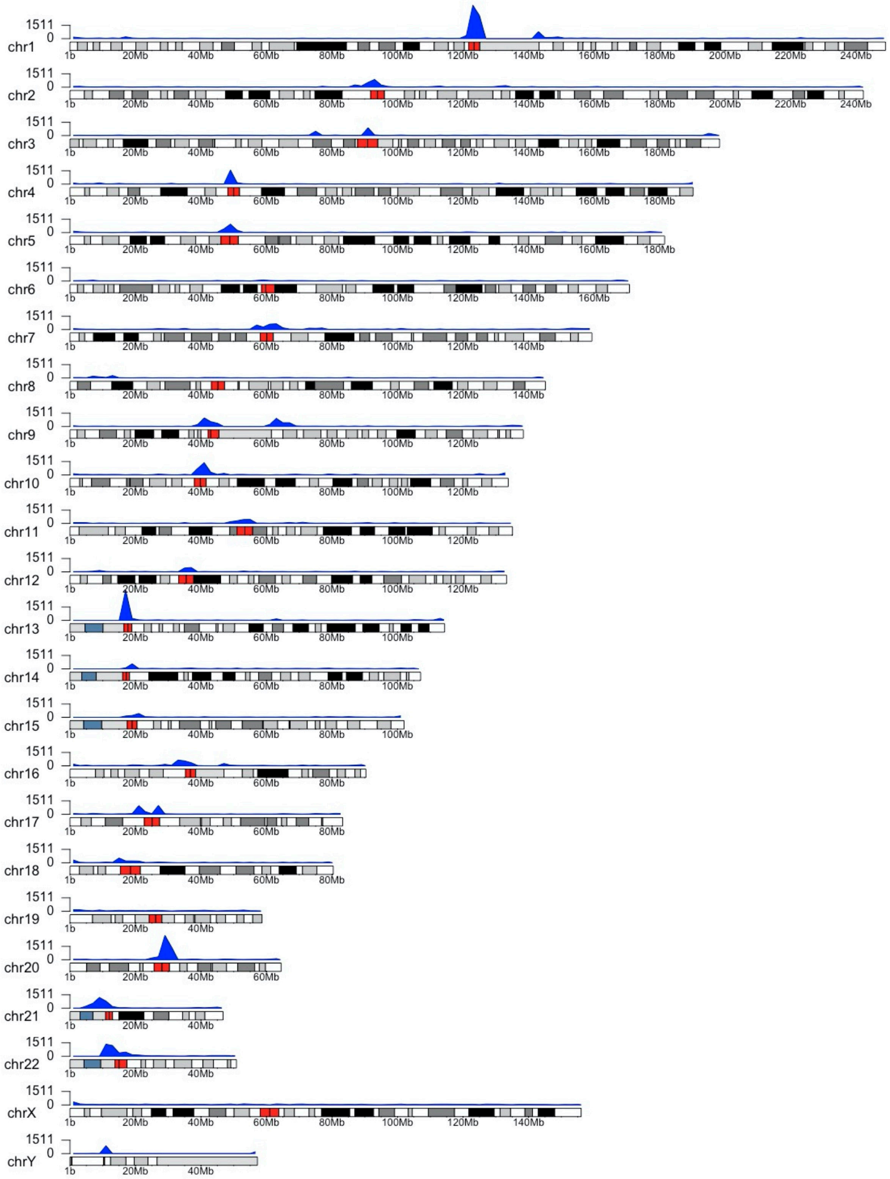

Figure S6. Disproportionate distribution of common mutations in genome: Karyoplot showing enrichment of mutations (blue peaks) in pericentromeric region (marked with red on cytoband) where genomic positions have been plotted against SNP density in corresponding regions using R package (karyoploteR 1.18.0).

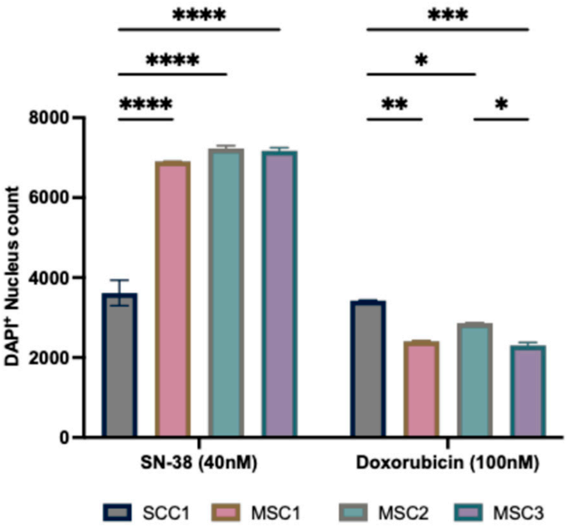

Figure S7. SN-38 resistant mutants retain sensitivity to doxorubicin: Cells were treated with SN-38(40nM) or doxorubicin (100nM) for 24 hours or left untreated. After 24 hours, cells were washed to remove dead cells and fixed, stained by DAPI. Experiment was conducted in biological triplicates (n=3). Quantification of data presented is the number of $DAPI^+$ nucleus count after treatment, n=183 images were analyzed with the integrated software (refer material and methods). Statistics were calculated using GraphPad Prism version 9.0.0, California USA. The significance of differences was determined using Two-way NAOVA (*P < 0.0332, **P < 0.0021, ***P < 0.0002, ****P <0.0001) denoted.

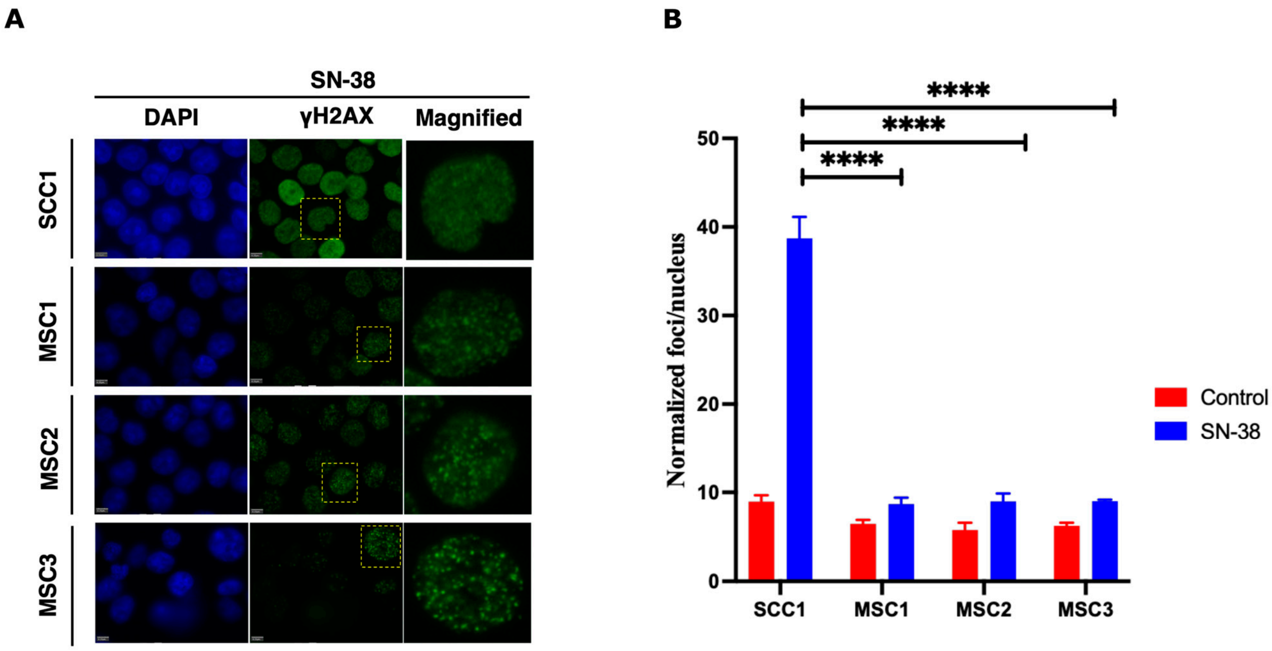

Figure S8. Resistant mutant experiences lower frequency of DSB following SN-38 exposure to short treatment. **(A)** γH2AX foci in cells exposed to SN-38 (40nM for 30min). SCC1 cells are compared to the mutants after very short treatment. SCC1 experiences significantly higher DSB compared to resistant clones, for long exposure data refer (Figure 9A-C). Experiment was conducted in biological triplicates. **(B)** Quantification of data presented in Fig. S9A of the number of foci generated 30-min post treatment, n=168 images captured at 40x, were analyzed with the integrated software (refer material and methods). Statistics were calculated using GraphPad Prism version 9.0.0, California USA. The significance of differences was determined using unpaired Welch's correction, two-tailed t-test (****P <0.0001) denoted in above in figure (B).

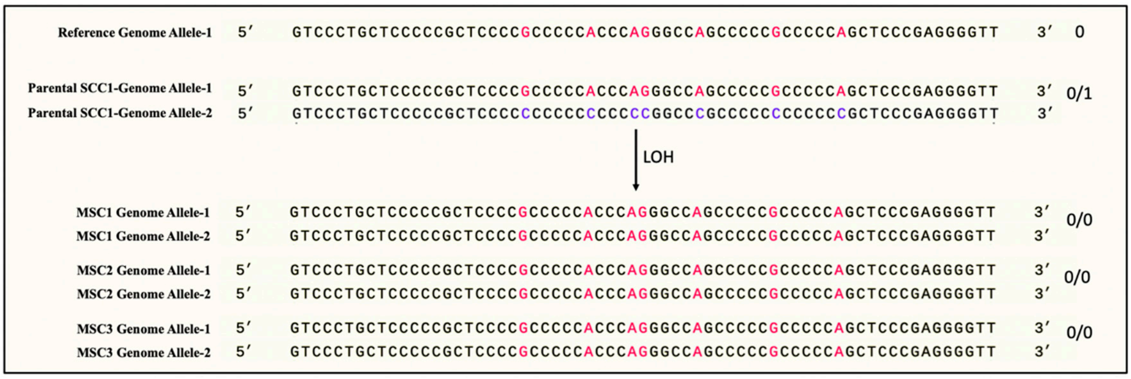

Figure S9. Representative example mutations in polyC sequences. Comparative alignment of a polyC sequence in SSC1 with reference genome (hg38) and resistant sub-clones. Allele-1 and Allele-2 represent two alleles in homologous chromosomes. Sequence marked by (both red) alleles represents homozygosity (0/0) as shown in reference genome strand and resistant sub-clones (top). Alleles (red and blue) in parental line represent heterozygosity (0/1) with "Cs" base. After the SN-38 adaptation, generated mutations lead to LOH, so that the polyC tract became interrupted as in normal reference genome alleles.
